# Risk factors and prognostic significance of early postoperative complications for patients who underwent pneumonectomy for lung cancer

**DOI:** 10.1186/s13019-024-02777-w

**Published:** 2024-05-03

**Authors:** Güntuğ Batıhan, Kenan Can Ceylan, Şeyda Örs Kaya

**Affiliations:** 1 Department of Thoracic Surgery, Kafkas University Medical Faculty, Sehitler district, Kars, 36100 Turkey; 2 Dr Suat Seren Chest Diseases and Chest Surgery Training, Research Hospital, University of Health Sciences Turkey, Izmir, Turkey

**Keywords:** Lung cancer, Pneumonectomy, Postoperative complication, Prognosis

## Abstract

**Background:**

Although pneumonectomy has relatively high mortality and morbidity rates, it remains valid in the surgical treatment of lung cancer. This study aims to evaluate the prognostic significance of postoperative complications after pneumonectomy and demonstrate the risk factors related to early postoperative complications.

**Methods:**

Patients who underwent pneumonectomy for non-small cell lung cancer between January 2008 and May 2021 were included in the study. Factors related to the development of early postoperative complications and overall survival were evaluated by univariate and multivariate analyses.

**Results:**

A total of 136 patients were included in the study. Early postoperative complications were seen in 33 (24.3%) patients and late postoperative complications in 7 (5.1%) patients. The amount of cigarette smoking, and the operation side were the independent variables that affect the development of early postoperative complications. In multivariate analysis, smoking amount and pericardial invasion were associated with the development of postoperative hemorrhage, and advanced age was associated with the development of postoperative pneumonia.

**Conclusions:**

Early postoperative complications have a negative effect on the prognosis after pneumonectomy therefore careful patient selection and preoperative risk assessment are essential to minimize the occurrence of complications and improve patient outcomes.

**Trial registration:**

This observational study was approved by the (Ethical Committee of Dr. Suat Seren Chest Diseases and Chest Surgery Education and Research Center) Institutional Review Board of our center (E-49109414-604.02.02-218625439).

## Background

Surgery has an important role in the treatment of lung cancer, which is responsible for 18% of cancer-related deaths worldwide [1]. Pneumonectomy is a surgical procedure in which the entire lung is removed, and can be applied in the surgical treatment of malign or benign lung diseases. Although less parenchymal loss is desirable in the surgical treatment of lung cancer, cases, where pneumonectomy is unavoidable, are not uncommon. Higher postoperative mortality and morbidity rates in pneumonectomy compared to other lung resections necessitate careful attention in patient selection [2, 3]. In our study, we aimed to analyze the data of patients who underwent pneumonectomy for non-small cell lung cancer and to reveal the prognostic significance of postoperative complications.

## Methods

### Ethical statement

This observational study was approved by the Institutional Review Board of our center. Informed consent was obtained from the patients included in the study.

### Patient selection

The data of the patients who underwent pneumonectomy for non-small cell lung cancer between January 2008 and May 2021 were collected retrospectively. Patients who had completion pneumonectomy, salvage surgery, and extended lung resection (lung resection with chest wall, diaphragm, tracheal carina, superior vena cava, atrium, or aorta) were not included in the study.

For preoperative pulmonary risk assessment, spirometry +/- diffusing capacity for carbon monoxide (DLCO) was performed for each patient. In patients with low FEV1 (%) lung perfusion scintigraphy was performed to calculate predicted postoperative FEV1. If necessary, VO2max and cardiopulmonary exercise tests were also performed. Thorax computed tomography (CT), cranial MR or CT, Positron emission tomography (PET-CT), and fiberoptic bronchoscopy were performed for all patients before surgery. The preoperative mediastinal staging was performed with endobronchial ultrasound-guided transbronchial needle aspiration (EBUS-TBNA) or mediastinoscopy in the presence of suspicious mediastinal lymph nodes on thorax CT or PET-CT. In the preoperative period, respiratory exercise training and nutritional support were provided to the patients. In addition, according to the presence of risk factors such as advanced age, a history of cardiac disease, and extracardiac comorbidity pharmacologic prophylaxis for postoperative atrial fibrillation was initiated.

Patients were placed in the lateral decubitus position. Muscle-sparing lateral thoracotomy or tri-portal video-assisted thoracoscopic surgery (VATS) was performed. Although we did not have strict rules in the selection of the operation method (VATS/Thoracotomy), we had some basic principles that matured over time with our increasing experience:

1. Because of the difficulty in removing the specimen from the intercostal space, thoracotomy was frequently preferred in the presence of tumor with a solid component larger than 5 cm in chest CT.
2. Thoracotomy was frequently preferred in centrally located tumors with suspected invasion of mediastinal structures (atrium, pulmonary trunk, tracheal carina).
3. If there was a possibility of parenchyma sparing with advanced surgical techniques (e.g., arterioplasty of pulmonary artery with pericardial patch, bronchovascular sleeve), thoracotomy was preferred.

The possibilities were evaluated to avoid pneumonectomy in all patients. In cases where parenchymal sparing methods were not possible in terms of oncological principles and/or surgical technique, pneumonectomy was decided. Three ports were used in cases where VATS was preferred. Automatic stapling devices were used in the majority of the cases. Resection margins of the main bronchus were confirmed via the frozen section and the underwater test with sustained airway pressure of 30–35 mmH2O was performed to control air leakage. Mediastinal lymph node dissection was performed following current guidelines. In patients, at high risk for bronchopleural fistula (right pneumonectomy, presence of neoadjuvant therapy, patients with poor nutritional status, and presence of diabetes) the bronchial stump was supported with a thymopericardial fat flap. A chest tube was placed in all cases at the end of the operation.

All patients had regular follow-up visits every 3 months for the first 2 years, then every 6 months up to 5 years. The data regarding the follow-up of the patients were obtained through the hospital records and direct telephone calls if needed.

### Definitions

Charlson Comorbidity Index was used to classify the comorbid conditions of patients. Postoperative complication was defined as any deviation from the normal postoperative course. Postoperative complications occurring within the first 30 days were classified as early postoperative complications, while clinical pathologies seen after 30 days and related to the surgical procedure were classified as late postoperative complications. Bleeding from the operation site and requiring re-operation (re-thoracotomy or re-VATS) was classified as postoperative hemorrhage. Postoperative pneumonia was defined as the presence of deterioration in respiratory parameters with the appearance of newly developed parenchymal infiltration radiologically in the postoperative period. All mortalities within the first 30 days postoperatively were classified as postoperative mortality.

### Statistical analysis

SPSS 25.0 (SPSS Inc., Chicago, IL, USA) was used to perform statistical analysis. The normality of distribution was tested with the Shapiro-Wilk test for all numerical variables. Chi-squared or Fischer’s exact tests were used to compare frequencies in categorical variables. Continuous variables are expressed as mean value ± standard deviation (SD) and discrete variables are expressed as numbers and percentages.

Kaplan-Meier method was used to estimate disease-free survival (DFS) and overall survival (OS). The difference in survival outcomes was evaluated with the Long-Rank test. Variables that may have an impact on postoperative complications and survival were determined by modeling based on clinical practice and literature data. These variables were evaluated with univariate and multivariate (Logistic regression and Cox regression analyses) analyses. Variables that were suggested to have an impact on prognosis in previous studies were included in univariate analyses. In logistic regression and cox-regression analyses backward elimination method was preferred to select variables and threshold p value was determined as 0.05. Thus, the variables included in the univariate analysis were gradually subtracted from the model so that the p-value of all variables remained below 0.05, and the final model was reached. Variables included in the Cox regression analysis were tested for multicollinearity, sample size, and outliers. Statistical significance was set at P-value < 0.05 (All P values presented were 2-sided).

## Results

From January 2008 to May 2021 a total of 136 patients were included in the study. The mean age was 62.2 ± 7.5 years. There were 119 male and 15 female patients. The characteristics of the patients are summarized in Table 1. Left pneumonectomy was performed in 105 patients and right pneumonectomy in 31 patients. Intraoperative complications were observed in 4 (2.9%) patients, including vascular injury in 2 and bronchial injury due to intubation in the other 2 patients. There was no intraoperative mortality. Early postoperative complications were seen in 33 (24.3%) patients and late postoperative complications in 7 (5.1%) patients. The most common early postoperative complication was pneumonia (10.3%), and the second most common was hemorrhage (6.6%). The pathologic results of the patients are presented in Table 2. N2 nodal metastases were detected in 28, and visceral pleural invasion was detected in 21 patients. The univariate and multivariate analyzes of the factors that may affect the development of postoperative complications are shown in Table 3. There was no correlation or collinearity that could cause bias between the variables included in the multivariate analysis.

**Table 1:** Patient characteristics

Characteristics	*N* = 136
**Age (years) (mean ± SD)**	62.2 ± 7.5
**Sex (male)**	119 (87.5%)
**Comorbidities**	51 (37.5%)
Diabetes mellitus	21 (15.4%)
Hypertension	9 (6.6%)
Coronary artery diseases	11 (8.1%)
Multiple	10 (7.4%)
**Smoking (yes)**	125 (93.2)
**Smoking amount (pack-year) (mean ± SD)**	42.8 ± 26.7
**FEV1 (lt) (mean ± SD)**	2.17 ± 0.55
**FEV1 (%) (mean ± SD)**	75.2 ± 15.6
**Preoperative serum albumin level (g/dl) (mean ± SD)**	4.18 ± 0.58
**Neoadjuvant therapy**	30 (22.1%)
Chemotherapy	25 (18.4%)
Chemoradiotherapy	5 (3.7%)
**Histology**	
Squamous cell carcinoma	93 (68.4%)
Adenocarcinoma	36 (26.5%)
Large cell carcinoma	7 (5.1%)
**Operation side**	
Right	31 (22.8%)
Left	105 (77.2%)
**Surgical approach**	
VATS	20 (14.7%)
Thoracotomy	116 (85.3%)
**Intraoperative complication**	4 (2.9%)
Major vascular injury	2 (1.5%)
Bronchial laceration	2 (1.5%)
**Operation time (minutes) (mean ± SD)**	190.8 ± 76.2
**Early postoperative complication**	33 (24.3%)
Pneumonia	14 (10.3%)
Hemorrhage	9 (6.6%)
Arrythmia	3 (2.2%)
Acute renal failure	2 (1.5%)
BPF	1 (0.7%)
Cerebrovascular accident	1 (0.7%)
ARDS	1 (0.7%)
Pulmonary embolism	1 (0.7%)
Chylothorax	1 (0.7%)
**Late postoperative complication**	
BPF +/- Empyema	7 (5.1%)
**Postoperative mortality (yes)**	9 (6.6%)
**Adjuvant therapy**	110 (80.9%)
Chemotherapy	87 (63.9%)
Radiotherapy	2 (1.5%)
Chemoradiotherapy	21 (15.4%)

SD: Standard deviation, FEV: Forced expiratory volume, ARDS: Acute respiratory distress syndrome, BPF: Bronchopleural fistula

**Table 2:** Pathological results

Characteristics	*N* = 136
Tumor size (cm) (mean ± SD)	5.3 ± 2.2
T stage	
1	24 (17.6%)
2	37 (27.2%)
3	46 (33.8%)
4	29 (21.3%)
N stage	
0	62 (45.6%)
1	46 (33.8%)
2	28 (20.6%)
TNM Stage	
I	14 (10.3%)
II	69 (50.7%)
III	53 (40.0%)
Bronchial margin (cm) (mean ± SD)	2.3 ± 1.6
Visceral pleural invasion	
PL0	101 (75.7%)
PL1	12 (8.8%)
PL2	21 (15.4%)
Pericardial invasion (yes)	27 (19.9%)

SD: Standard deviation

**Table 3:** Analyzing risk factors for early postoperative complications

		Early postoperative complication				Postoperative hemorrhage				Postoperative pneumonia			
		Univariate analysis		Multivariate analysis		Univariate analysis		Multivariate analysis		Univariate analysis		Multivariate analysis	
Variables	**Ref.**	**p value**	**Odds ratio**	**p value**	**Odds ratio**	**p value**	**Odds ratio**	**p value**	**Odds ratio**	**p value**	**Odds ratio**	**p value**	**Odds ratio**
Age (years)		0.83	0.57	-	-	0.93	1.00	-	-	0.086	1.08	0.044	1.10
Comorbidity (yes)	No	0.68	0.85	-	-	0.40	0.57	0.066	1.08	0.22	2.14	-	-
Smoking amount (pack-year)		0.048	1.05	0.049	1.01	0.028	1.02	0.042	1.03	0.42	1.00	-	-
FEV1 (%)		0.47	0.99	-	-	0.08	0.96	0.065	0.93	0.95	1.00	-	-
Neoadjuvant treatment (yes)	No	0.66	1.22	-	-	0.36	0.37	-	-	0.43	1.66	-	-
Preoperative serum albumin		0.059	0.47	0.076	0.46	0.56	0.69	-	-	0.45	0.65	-	-
Operation side (right)	Left	0.009	3.10	0.010	3.26	0.044	3.85	-	-	0.17	2.33	-	-
Surgical approach (VATS)	Thoracotomy	0.22	0.44	0.11	0.32	0.67	0.63	-	-	0.94	1.06	-	-
Tumor size (cm)		0.58	1.05	-	-	0.79	1.04	-	-	0.62	0.94	-	-
Pleural invasion (yes)	No	0.37	0.65	-	-	0.71	0.73	-	-	0.16	0.23	0.15	0.21
Pericardial invasion (yes)	No	0.082	2.20	-	-	0.004	7.21	0.002	15.58	0.26	0.29	0.14	0.19
Advanced stage (stage III)	Stage I-II	0.57	1.30	-	-	0.92	1.07	-	-	0.86	0.90	-	-
Operation time (minutes)		0.18	1.01	-	-	0.44	0.99	-	-	0.44	1.00	-	-

FEV1: Forced expiratory volume in the first second. Ref: Reference

The amount of cigarette smoking, and the operation side were the independent variables that affect the development of early postoperative complications.

In multivariate analysis, smoking amount and pericardial invasion were associated with the development of postoperative hemorrhage, while advanced age was associated with the development of postoperative pneumonia (Table 3).

Postoperative mortality was observed in 9 (6.6%) patients. In 4 of these patients, mortality was observed after reoperation due to postoperative hemorrhage. Other causes of postoperative mortality were pneumonia in 3 patients, BPF in 1 patient, and ARDS in 1 patient.

**Fig. 1:**
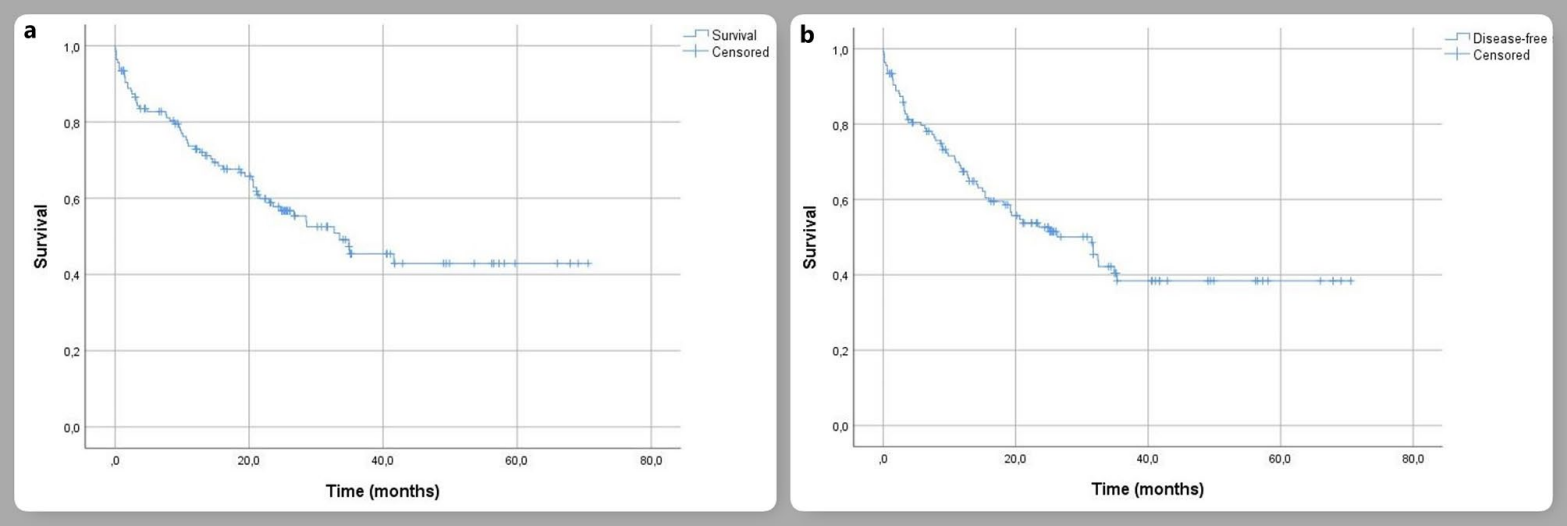
**a** indicates Kaplan-Meier survival curve for overall survival. **b** indicates Kaplan-Meier survival curve for disease-free survival

**Fig. 2:**
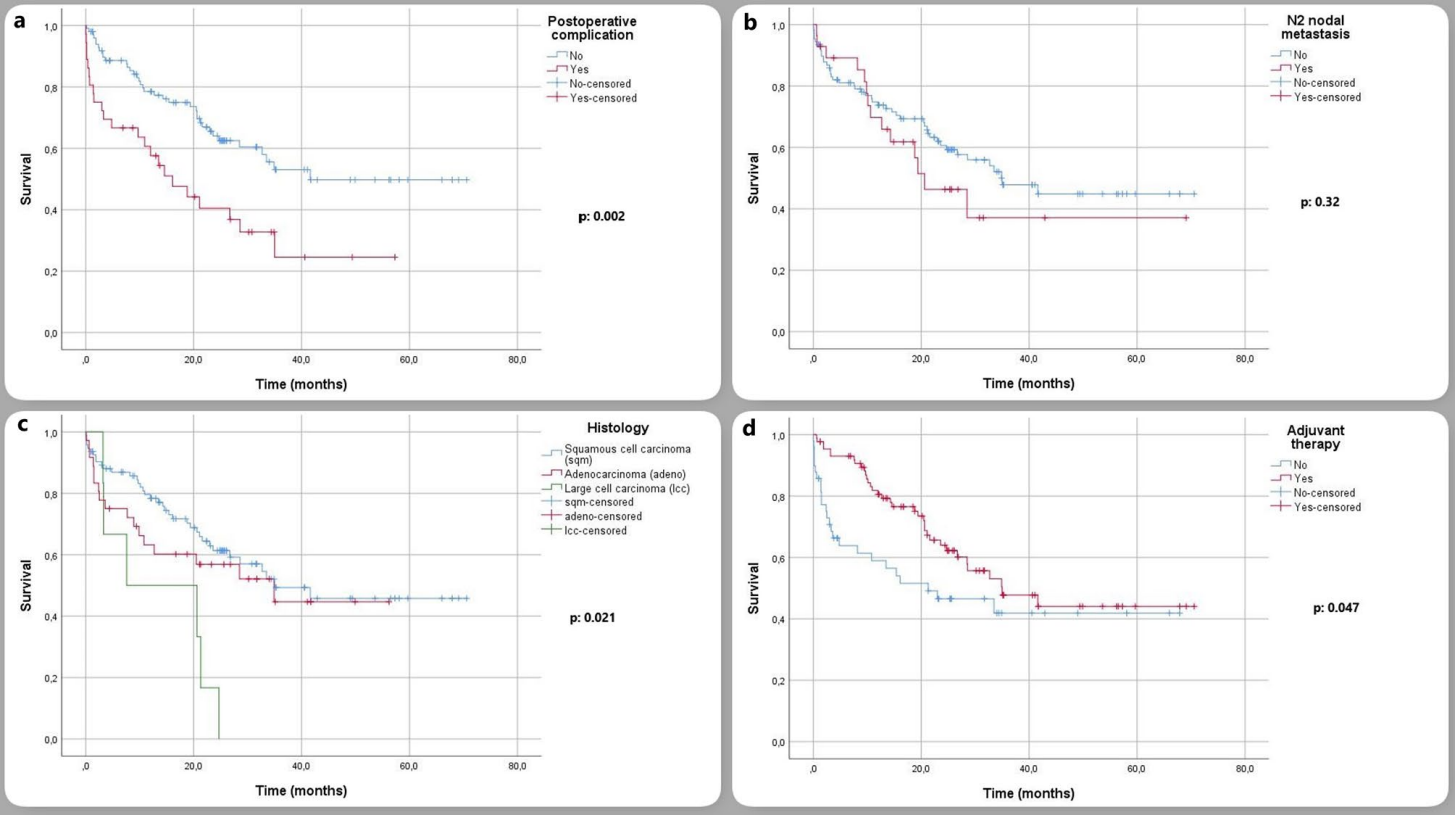
In this figure, survival curves based on postoperative complications (**a**), N2 nodal metastases (**b**), histology (**c**), and adjuvant treatment (**d**), respectively, are indicated

The mean follow-up was 21.5 ± 17.6 months and the mean overall survival was 39.3 ± 2.9 months. Disease-free survival was 35.7 ± 2.9 months. The 1, 3, and 5-year survival rates were 72.9 ± 3.9% and 45.4 ± 5.4, respectively (Fig. 1). In univariate analysis, histological subtype, tumor size, presence of postoperative complications, and adjuvant therapy were found to be prognostic factors affecting survival (Fig. 2). In multivariate analysis, operation side, histological subtype, N2 nodal metastasis, presence of postoperative complications, and adjuvant treatment were found to be independent prognostic factors (Table 4).

**Table 4:** Prognostic factors for survival

		Univariate analysis			Multivariate analysis		
Variables	**Ref.**	**p value**	**Hazard ratio**	**95% CI**	**p value**	**Hazard ratio**	**95% CI**
Age (years)		0.314	1.02	0.98–1.06	0.061	1.03	0.99–1.07
Comorbidity (yes)	No	0.73	1.09	0.65–1.83	-	-	-
Smoking amount (pack-year)		0.25	0.99	0.98-1.00	-	-	-
Operation side (right)	Left	0.065	1.68	0.97–2.92	0.049	1.80	1.01–3.23
Histology		0.021			0.01		
Adenocarcinoma	Squamous cell ca.	0.407	1.28	0.72–2.27	0.12	1.64	0.89–3.04
Large cell carcinoma		0.006	3.43	1.43–8.21	0.00	6.03	2.35–15.46
Tumor size (cm)		0.003	1.18	1.06–1.32	-	-	-
Pleural invasion (yes)	No	0.76	1.09	0.62–1.94	-	-	-
Pericardial invasion (yes)	No	0.73	1.11	0.80–2.06	-	-	-
Bronchial margin (cm)		0.69	1.03	0.88–1.21	-	-	-
N2 nodal metastasis (yes)	No	0.32	1.36	0.74–2.48	0.029	2.06	1.08–3.94
Early postoperative complication (yes)	No	0.002	2.24	1.33–3.78	0.00	2.91	1.63–5.17
Adjuvant therapy (yes)	No	0.047	0.59	0.36–0.99	0.03	0.43	0.25–0.75

Disease recurrence was observed in 35 (25.7%) of the patients during follow-up. The most common cause of disease recurrence was distant metastasis, and the brain was the most common location (n:12, 8.8%).

## Discussion

While minimizing parenchymal loss is preferred in the surgical treatment of lung cancer, there are cases where pneumonectomy becomes unavoidable. The higher rates of postoperative mortality and morbidity associated with pneumonectomy compared to other lung resections highlight the importance of careful patient selection [4–6]. This study aimed to emphasize the prognostic importance of the development of postoperative complications in patients who underwent pneumonectomy and to examine the risk factors affecting its development. Our findings highlight the significance of smoking amount and operation side as an independent risk factor for developing early postoperative complications. In addition, the amount of smoking and pericardial invasion were found to be associated with postoperative hemorrhage and advanced age with postoperative pneumonia.

In our study, the development of postoperative complications was found to be associated with poor prognosis. The relationship between the development of postoperative complications and poor prognosis after lung resections has been demonstrated in different studies [7–10]. It is well recognized that postoperative complications can prolong hospital stay, delay recovery, and increase the risk of mortality. The occurrence of complications after lung resection can lead to prolonged inflammation, impaired wound healing, and compromised pulmonary function. These factors may contribute to a higher risk of disease recurrence and decreased overall survival rates. This hypothesis was also supported in various studies in which the development of postoperative complications was considered as a prognostic factor. Lugg et al. [11] revealed the relationship between the development of postoperative complications and poor prognosis and explained this situation with the increased rate of respiratory dysfunction in the patient group with postoperative complications. In other studies, Alifano et al. [12] and Shinohara et al. [13] emphasized the relationship between C-reactive protein, interleukin-6, and tumor necrosis factor-α levels, which expect to increase in the presence of surgery-related complications, with cancer progression. However, there are few studies examining the postoperative complication-prognosis relationship specific to pneumonectomy [14, 15]. Alloubi et al. [14] emphasized that the development of postoperative complications after pneumonectomy is associated with high mortality and that maximum attention should be paid in cases of advanced age and heart failure. In another study, Gu et al. found no association between postoperative complications and overall or recurrence-free survival [15].

In previous studies many different risk factors like advanced age, high ASA physical status, chronic obstructive pulmonary disease, coronary artery disease, diabetes, right pneumonectomy, and smoking have been focused on the development of postoperative complications after pneumonectomy [14, 16, 17]. The possible pathogenesis of tobacco smoking, which was also found to be effective on the development of postoperative complications in our study, is well known. Increased secretion production, decreased macrophage function, decreased ciliary motility, and increased serum carboxyhemoglobin levels are known effects of smoking on the respiratory system [18, 19]. With these mechanisms, smoking can cause an increase in postoperative pulmonary complications. In addition to studies revealing the risks created by the cumulative effect of smoking, there are also studies emphasizing the positive effects of smoking cessation before the operation, regardless of the amount of previous smoking [20]. Although we could not reveal the effect of smoking cessation before the operation due to insufficient data in our study, we were able to show that the amount of past smoking had an effect on the development of postoperative complications.

In our study, right pneumonectomy was found to be an independent risk factor for postoperative complications. This situation has also been demonstrated in similar studies, and the higher alveolar reserve of the right lung and the anatomically more sheltered localization of the left hilum have been suggested as possible causes [21].

Another postoperative complication we would like to emphasize is hemorrhage. In our study, 9 patients were reoperated for postoperative hemorrhage, and the amount of smoking and pericardial invasion were found to be independent risk factors. Studies in the field of thoracic surgery focused on the effect of smoking in terms of postoperative respiratory complications. However, few studies from other surgical branches have reported that smoking increases the rates of postoperative bleeding, referring to its demonstrated negative effects on the coagulation cascade and platelet aggregation [22, 23].

Although intrapericardial pneumonectomy has been associated with increased the risk of postoperative mortality, tachyarrhythmia and cardiac herniation, no study associated with postoperative hemorrhage has been found [24]. Missing a bleeding focus on the pericardial dissection site or performing a more aggressive lymph node curettage in these cases can be considered as possible causes of postoperative hemorrhage.

Pneumonia was the most common major postoperative complication in most series [25, 26]. Advanced age, male gender, atelectasis, and smoking have been reported as risk factors for post-pneumonectomy pneumonia [4–6, 25, 26]. Consistent with the literature data, pneumonia was the most common postoperative complication in also our series with a rate of 10.3% and advanced age was found as an independent risk factor (OR: 1.10; P: 0.044).

This study has several limitations that should be acknowledged. First, the retrospective nature of the study design introduces inherent limitations, such as potential selection bias and incomplete data collection. The reliance on medical records and available data may have resulted in missing or incomplete information, which could impact the accuracy and generalizability of the findings.

Secondly, the sample size in this study was relatively small, which might limit the statistical power and generalizability of the results. A larger sample size and multi-center studies would provide more robust and representative findings.

Thirdly, this study was conducted at a single institution, which may limit the generalizability of the findings to other healthcare settings and populations. The variations in surgical techniques, perioperative care, and patient characteristics across different institutions may influence the occurrence and management of postoperative complications.

Lastly, this study primarily examined early postoperative complications and their prognostic importance. Long-term outcomes and survival were not extensively investigated.

Despite these limitations, this study provides valuable insights into the prognostic importance of postoperative complications following pneumonectomy for lung cancer. Future research with larger sample sizes, prospective designs, and multi-center collaborations is needed to validate and expand upon these findings.

## Conclusions

In conclusion, our study underscores the prognostic significance of early postoperative complications in patients underwent pneumonectomy for lung cancer. Smoking amount, operation side, pericardial invasion and advanced age were identified as independent risk factors for early postoperative complications, including hemorrhage and pneumonia. These findings highlight the importance of careful patient selection and preoperative risk assessment to minimize the occurrence of complications and improve patient outcomes. It is crucial for healthcare providers to consider these risk factors and take appropriate measures to mitigate complications in pneumonectomy patients. Meticulous surgical techniques, vigilant postoperative care, and early recognition and management of complications, particularly in high-risk patients, are essential to reduce the burden of postoperative complications and enhance patient recovery.

## Data Availability

No datasets were generated or analysed during the current study.
